# Aiming higher for maternal and child nutrition in South Asia

**DOI:** 10.1111/mcn.12739

**Published:** 2018-11-29

**Authors:** Harriet Torlesse, Víctor M. Aguayo

**Affiliations:** ^1^ Nutrition Section UNICEF Regional Office for South Asia Kathmandu Nepal; ^2^ Nutrition Section, Programme Division UNICEF New York New York

**Keywords:** anaemia, child feeding, early childhood development, maternal nutrition, South Asia, stunting, wasting

## Abstract

The sustainable development of nations relies on children developing to their full potential and leading healthy, productive, and prosperous lives. Poor nutrition in early life threatens the growth and development of children, especially so in South Asia, which has the highest burdens of stunting, wasting, and anaemia in the world. Targeted actions to reduce stunting and other forms of child malnutrition in South Asia should be informed by an understanding of what drives poor nutrition in children, who is most affected, and effective programme approaches. To this end, the UNICEF Regional Office for South Asia commissioned a series of papers in 2016–2017 to fill knowledge gaps in the current body of evidence on maternal and child nutrition in South Asia, including analyses of: (a) the links between anthropometric failure in children and child development; (b) the time trends, current distribution, disparities and inequities of child stunting, wasting and anaemia, and their direct and underlying causes, including maternal anaemia, low birth weight, breastfeeding, and complementary feeding; (c) policy and programme actions to increase the coverage of nutrition interventions during pregnancy, improve breastfeeding practices, and care for severely wasted children. This overview paper summarizes the evidence from these analyses and examines the implications for the direction of future advocacy, policy, and programme actions to improve maternal and child nutrition in South Asia.

**Key messages**

- The South Asia region bears a disproportionate burden of stunted children who have worse health, cognition, and learning outcomes.
- Stunted children are concentrated in the more socio‐economically disadvantaged households, and often experience multiple forms of nutrition deprivation, including low birth weight, stunting, wasting, and anaemia.
- Poor maternal nutrition (low body mass index, short stature, and anaemia), low birth weight and poor diets in infancy and early childhood are consistent predictors of stunting and wasting.
- A coordinated approach involving the food, health, and social protection systems is needed to improve the diets of women and children in early life, while girls' access to educational opportunities and households' access to and use of safe water and sanitation services is scaled up.

## 1 INTRODUCTION

South Asia's policymakers have important decisions to make to accelerate economic growth and prosperity in their countries. There is mounting global evidence that good nutrition in early life has enduring positive consequences for cognitive development, school readiness, learning outcomes, human capital formation, economic productivity, and shared prosperity (Shekar, Kakietek, Eberwein, & Walters, 2017; UNICEF, 2018; Victora et al., 2008). This explains why investments in nutrition are considered central to the achievement of the Sustainable Development Goals (SDG) (Development Initiatives, 2017).

Latest global estimates indicate that almost 40% of the world's stunted children (59 million children) and 53% of the world's wasted children (27 million children) live in South Asia (UNICEF et al., 2018). These children experience worse health and developmental deficits in childhood and lower productivity and poorer livelihoods in adulthood, leading to losses of human capital (Black et al., 2013). Although the prevalence of child stunting is falling in the region (UNICEF et al., 2018), the pace of progress is too slow and most countries with available data are not on track to meet the stunting reduction target or other nutrition targets of the SDGs (Development Initiatives, 2017). Further, the Sustainable Development Agenda has a quest to “leave no‐one behind” (UNDP, 2018), and so national‐level progress to improve nutrition is insufficient unless inequalities are also resolved.

Targeted actions to reduce stunting and other forms of malnutrition should be informed by an understanding of what drives poor nutrition in children and who is most affected. Recent evidence suggests that the poor nutrition of women before and during pregnancy, the poor diets of young children, and poor sanitation practices are common drivers of restricted linear growth in South Asia (Aguayo & Menon, 2016; Kim, Mejia‐Guevara, Corsi, Aguayo, & Subramanian, 2017). However, drivers vary contextually and over time and need to be periodically assessed (Aguayo & Menon, 2016).

In 2016–2017, the UNICEF Regional Office for South Asia commissioned a series of analyses to fill key knowledge gaps in the current body of evidence on maternal and child nutrition in South Asia. This series includes analyses of the links between anthropometric failure in children and child development; the time trends, current distribution, disparities and inequities of child stunting, wasting and anaemia; their direct and underlying causes, including maternal anaemia, low birth weight (LBW), breastfeeding and complementary feeding; and policy and programme actions to increase the coverage of nutrition interventions during pregnancy, improve breastfeeding practices and care for severely wasted children. The 15 articles included in this special issue of Maternal and Child Nutrition summarize the findings of these analyses.

## 2 CHILD GROWTH AND DEVELOPMENT

South Asia's most disadvantaged children face the greatest challenges in being well‐nourished. Research indicates that children in the poorest wealth quintile are more likely to be stunted in every country in the region (Rama, Béteille, Li, Mitra, & Newman, 2015). In this issue, Krishna, Mejía‐Guevara, McGovern, Aguayo, and Subramanian (2018) analyse socio‐economic inequalities and trends in child stunting in South Asia between 1991 and 2014 using national survey data from Bangladesh, India, Nepal and Pakistan. Analysis of pooled data from all four countries shows that stunted is concentrated among children of households experiencing multiple forms of deprivation, including poor child diets, low levels of maternal education and household poverty. They also find large inter‐country differences in average rates of stunting reduction, ranging from 0.6 percentage points (pp) per year in Pakistan to 1.3 pp in India, 2.9 pp in Bangladesh, and 4.1 pp in Nepal. Stunting has declined across all wealth quintiles in all countries; however, the inequalities among wealth quintiles have largely persisted over time and have widened in both Nepal and Pakistan.

Focusing on Bhutan, Kang, Aguayo, Campbell, Dzed, et al. (2018) also examine the trends and determinants of stunting using national survey data. The average rate of reduction in stunting prevalence increased from approximately 1.3 pp per year between 1986/1988 and 2008 to 1.96 pp per year in the subsequent 7 years (2008 to 2015), giving an average reduction rate of 1.43 pp over the entire period. Given the low current prevalence of wasting in this population (2.5%), the authors suggest that factors other than energy deficit are limiting linear growth. However, the analysis of determinants does not uncover any significant predictors of stunting besides older child age, possibly because of the relatively low prevalence of stunting (21%) and small survey sample size.

Evidence suggests that wasting adversely affects the linear growth of children, indicating that the early identification and treatment of wasting may play a role in stunting prevention efforts (Khara & Dolan, 2014). In their paper, Aguayo et al. (2018) conduct a retrospective case series analysis to examine the effectiveness of Pakistan's community management of acute malnutrition programme. Most (87%) severely wasted children were aged 6–23 months, the age range during which there is a steep increase in the percentage of stunted children in Pakistan. The programme was effective in achieving high survival (99.6%) and recovery (87.8%) rates among admitted children, meeting the international standards of care. Children with poorer anthropometry at admission (severely wasted or stunted) had higher death rates and lower recovery rates than other children. The authors conclude that prioritizing children aged 6–23 months and those with multiple anthropometric failure will increase the population‐level impact of the programme.

As child survival continues to improve in South Asia, the developmental consequences of poor nutrition in early life become a more pressing concern than mortality. Alongside the very high burdens of stunting and wasting, the region has the second highest number (27.8 million) of children with low cognitive and socioemotional test scores after the sub‐Saharan Africa region (McCoy et al., 2016). Kang, Aguayo, Campbell, and West (2018) examine pooled data from Multiple Indicator Cluster Surveys in Bangladesh, Bhutan, Nepal, and Pakistan and find that stunted children are at an increased risk of suboptimal learning/cognition development at 3–4 years, as measured by the Early Childhood Development Index. They find no association between wasting and learning/cognition development, possibly because the prevalence of wasting was low compared with stunting in the child population. The authors conclude that interventions that are effective in improving linear growth in the first years of life may also have positive dividends for early childhood development.

Childhood anaemia is also associated with impaired cognitive development, and possibly with motor development (Sachdev, Gera, & Nestel, 2005). Two studies in this special issue examine predictors of childhood anaemia: Harding, Aguayo, Namirembe, and Webb (2018) examine predictors in Nepal and Pakistan and Campbell et al. (2018a) examine predictors in Bhutan. The likelihood of anaemia is higher in children with an anaemic mother in Nepal and Pakistan and in infants compared with older children in all three countries, suggesting that mother's anaemia status may affect that of her child. Anaemia is also more common in stunted children in all three countries and in children with thin mothers in Pakistan, possibly reflecting the contribution of dietary inadequacy before and during pregnancy and in childhood. Children belonging to households in the poorest wealth quintiles in Pakistan and without an improved water facility in Nepal were more likely to be anaemic, reinforcing the need to target children from the most disadvantaged households.

## 3 MATERNAL NUTRITION AND LBW

Women's nutritional status, both before and during pregnancy, has a profound effect on foetal growth and development as well as the mother's own health and well‐being. A child whose pregnant mother is short, thin, or anaemic is more likely to experience in utero growth restriction, preterm delivery and LBW, and adverse outcomes for the mother include dystocia (difficult labour) and haemorrhage (Black et al., 2013).

South Asia has the highest prevalence of LBW (26%) of all regions in the world (Lee et al., 2013), reflecting the poor status of women's nutrition in the region. Using data from the most recent national demographic, health, and nutrition surveys, Goudet, Murira, Torlesse, Hatchard, and Busch‐Hallen (2018) estimate that one in 10 South Asian women of reproductive age have a low stature (<145 cm) and one in five have a low body mass index (<18.5 kg/m^2^), and overweight is rising rapidly. Both low stature and low body mass index in women have previously been identified as consistent predictors of child stunting in South Asian countries (Kim et al., 2017). In this issue, Harding, Aguayo, and Webb (2018) examine predictors of wasting and severe wasting using pooled national survey data from six South Asian countries. They find that children with reported LBW are significantly more likely to be wasted and severely wasted than non‐LBW children. In addition, LBW is a predictor of being simultaneously wasted and stunted.

There has been little progress in resolving maternal anaemia in South Asia (Stevens et al., 2013), and only Bangladesh seems to be on track to meet the World Health Assembly target to reduce maternal anaemia by 40% by 2025. In 2011, an estimated 47% of non‐pregnant women and 52% of pregnant women were anaemic, one of the highest prevalence levels of any region (Stevens et al., 2013). In their paper, Harding et al. (2018) examine the determinants of anaemia in women of reproductive age in Nepal and Pakistan and find it is associated with thinness (BMI <18.5 kg/m^2^) in both countries. They also find that children aged less than 5 years are more likely to be anaemic if their mother is also anaemic. Together with the findings of similar analyses conducted by Campbell et al. (2018a) in Bhutan, it is clear that anaemia is concentrated in the most disadvantaged women, including women from the poorest households in Pakistan, women without schooling in Bhutan, and women lacking sanitation facilities in Bhutan and Nepal. The prevalence of anaemia in pregnant women in Bhutan is lower than non‐pregnant, an atypical finding suggesting that iron folic acid supplementation during antenatal care is effectively protecting pregnant women from anaemia.

The high prevalence and co‐occurrence of maternal thinness, anaemia, and LBW in South Asia and their links with stunting, wasting, and anaemia in children suggests the need for greater policy and programme attention to improving women's nutrition to prevent these interrelated conditions. A combination of nutrition‐specific and nutrition‐sensitive actions is needed to address women's low quality diets, poor access to health and nutrition services, early age at marriage and pregnancy, low education, inadequate decision‐making power, and poor control over resources (Vir, 2016).

Focusing on nutrition‐specific interventions during pregnancy, Goudet et al. (2018) conduct a systematic review of recent evidence (2000–2017) on the barriers to and programme approaches for improving the coverage of maternal nutrition interventions in South Asia. The authors find evidence on a range of barriers acting at maternal (low women's education level and knowledge), household (low husband's education level, support from husband, and household wealth), and health facility level (late timing of first antenatal visit and low number of visits) that reduce the likelihood of pregnant women receiving and consuming iron folic acid and calcium supplements. The body of evidence on the effectiveness of programme approaches to improve the coverage of maternal nutrition interventions in South Asia is very small and largely focussed on only iron folic acid and calcium supplementation. Review of the nine included studies reveal that programme delivery platforms reaching pregnant women with supplements in their homes and communities, combined with information and counselling, can improve the access to services and consumption of supplements, particularly when the following conditions are met: (a) formative research is used to inform the programme design; (b) influential family members are engaged and support women; (c) the capacities, supervision and motivation of frontline‐workers are strengthened; and (d) there are no breaks in supply of supplements to service delivery points.

## 4 BREASTFEEDING

The benefits of breastfeeding on child survival, life‐long health, and cognitive development are well established (Victora et al., 2016). Evidence presented in this special issue indicates that optimal breastfeeding practices may also protect against child wasting and overweight in early childhood. Using pooled data from six South Asian countries, Harding, Aguayo, and Webb (2018) find that children are less likely to be wasted if they were breastfed within the first hour of birth, were not given any prelacteal foods, and are exclusively breastfed. The rapid fall in the prevalence of wasting during the first few months of life in several South Asian countries suggest that early and exclusive breastfeeding may help infants to recover from a low weight at birth. Focusing on Bhutan, Campbell et al. (2018b) report that children under 2 years are less likely to be overweight if they are currently breastfed.

The rates of optimal breastfeeding practices in the South Asia region are amongst the highest in the world (UNICEF, 2016), but progress has been uneven and practices are still not at desired levels. In their paper, Benedict, Craig, Torlesse, and Stoltzfus (2018a) examine time trends in breastfeeding practices between 1990 and 2016 in the five largest countries in South Asia and find that there has been a steady increase in the early initiation of breastfeeding, avoidance of prelacteal feeding, and exclusive breastfeeding in Bangladesh, India, and Nepal over the last 25 years. Despite these improvements, only about half of children in these countries benefit from the early initiation of breastfeeding and exclusive breastfeeding, and the rates of continued breastfeeding at 2 years have remained stagnant at ~70%. Progress in Afghanistan and Pakistan has lagged behind other countries in the region, with recent declines in all these breastfeeding practices in Afghanistan, as well as the early initiation of breastfeeding and avoidance of prelacteal feeding in Pakistan.

Nguyen et al. (2018) further explore breastfeeding trends in India to determine whether equity gaps have changed in recent years. Using data from national surveys, they report that socio‐economic inequalities in the early initiation of breastfeeding and exclusive breastfeeding narrowed between 2006 and 2016, a significant achievement given the rising economic equalities in the country. They find indicative evidence that the improvements in breastfeeding in the lower socio‐economic quintiles were driven by improved access to and use of health and nutrition services by mothers and children.

An understanding of what predicts suboptimal feeding and what works to improve breastfeeding practices can help to better target advocacy, policy, programme, and research efforts in the region. Two papers by Benedict et al. (2018a) and Benedict, Craig, Torlesse, and Stoltzfus (2018b) examine these issues. Multivariate analysis of national survey datasets from South Asia's five largest countries (Afghanistan, Bangladesh, India, Nepal, and Pakistan) reveal that the initiation of breastfeeding is more likely to be delayed in infants who were born by caesarean section (Benedict et al., 2018a). Common predictors of delayed initiation of breastfeeding, prelacteal feeding, and not being exclusively breastfed include a small size a birth, home delivery, and low women's empowerment. These findings suggest that infants born small or by caesarean section and their mothers need special breastfeeding support, and that programme approaches should be designed in the context of women's low empowerment in the region. Country‐specific predictors also exist, and need to be taken into consideration in the design of policies and programmes. In their scoping review of 31 studies (randomized trials and quasi‐experimental designs) on the effectiveness of programme approaches to improve breastfeeding practices in the region, Benedict et al. (2018b) report two key findings: first, programmes are more likely to be effective if they include multiple interventions (education and counselling, community mobilization, mass media, and newborn health initiatives such as kangaroo care and skin to skin contact) and involve multiple intervention environments (home/family, community, and health facility) to increase the opportunities to reach women and other family members and reinforce the promotion and support of breastfeeding; and second, the intervention coverage, timing relative to the age of the child, frequency, duration and targeting influence the effectiveness and should be carefully considered in the design of programme interventions.

## 5 COMPLEMENTARY FOODS AND FEEDING PRACTICES

When children reach 6 months of age, breastmilk no longer fully meets their nutrient needs, and dietary intake from complementary foods becomes a dominant influence on their growth and development. Poor complementary feeding practices are highly prevalent in South Asia (UNICEF, 2016) and often predict stunting and wasting in the first 2 years of life. An analysis of pooled data from five South Asian countries found that the likelihood of stunting is higher in infants aged 6–8 months who are not fed any complementary foods and in children aged 6–23 months whose diets do not meet minimum dietary diversity, defined in the analysis as daily diets that include at least four out of seven food groups (Kim et al., 2017). In this issue, Harding, Aguayo, and Webb (2018) report that children aged 6–23 months are more likely to be wasted if their diets do not meet the minimum dietary diversity and severely wasted if they are not fed enough meals (minimum meal frequency). In India, not meeting the minimum dietary diversity is also associated with the co‐occurrence of both being stunted and wasted (Harding, Aguayo, & Webb, 2018), a highly vulnerable state that carries a similar mortality risk to severe wasting (McDonald et al., 2013). In contrast, Campbell et al. (2018b) find that complementary feeding practices are not associated with stunting or wasting in Bhutan, possibly because the prevalence of anthropometric failure is relatively low in this population.

The majority of children in South Asia are not fed complementary foods according to the globally recommended practices. A review of national survey data (2006–2013) found that only 57% of infants aged 6–8 months are fed any complementary foods (the “timely introduction of complementary foods”), and among children aged 6–23 months, only 48% are fed with a minimum frequency, 33% are fed diets that meet the minimum dietary diversity, and 21% are fed diets that meet the minimum adequacy (sufficient number of meals, food groups, and breastmilk or milk feeds) (Aguayo, 2017). Across all countries, the minimum dietary diversity is consistently lower than minimum meal frequency, indicating that dietary diversity is a greater problem than frequency. Considerable variation in feeding practices exists between countries, and only Sri Lanka and the Maldives have rates that exceed 50% for all these practices.

Three studies in this special issue explore the predictors of complementary feeding practices by conducting multivariate analysis of national survey data from countries in the region, including Na, Aguayo, Arimond, Mustaphi, and Stewart (2018) in Afghanistan, Na, Aguayo, Arimond, Narayan, and Stewart (2018) in Bangladesh, and Na, Aguayo, Arimond, Dahal, et al., (2018) in Nepal. Together with a previously published study in Pakistan (Na, Aguayo, Arimond, & Stewart, 2017), the analyses show that complementary feeding practices are more likely to be suboptimal among infants (6–11 months), children who are first born, children whose mothers are younger or less educated, and in communities with poor access to health and nutrition services in these countries. Na, Aguayo, Arimond, Dahal, et al., (2018) find that Nepalese children with perceived LBW are also fed less diverse diets, which may stem from beliefs that they are too weak to digest some types of food; similar findings have been reported in Pakistan (Na et al., 2017). In addition, Na, Aguayo, Arimond, Dahal, et al., (2018) report that cultural beliefs continue to be a barrier to recommended feeding practices in Nepal, where the Dalit and minority ethnic and religious castes have poorer complementary feeding practices than other population groups.

Recent studies have found that children living in food insecurity or the poorest (lowest wealth quintile) households have less diverse diets in India (Chandrasekhar, Aguayo, Krishna, & Nair, 2017) and Pakistan (Na et al., 2017). Na, Aguayo, Arimond, Mustaphi, et al., (2018), Na, Aguayo, Arimond, Narayan, et al., (2018), and Campbell et al. (2018b) report an association between wealth quintile and dietary diversity in Afghanistan, Bangladesh, and Bhutan, respectively, suggesting that the availability of affordable nutritious foods is a common barrier to diverse diets in South Asian countries. Nguyen et al. (2018) show that the equity gaps in complementary feeding practices between socio‐economic status quintiles in India narrowed between 2006 and 2016, but practices remain poor across all groups.

Harmful feeding practices during childhood illness are a concern in South Asia. An analysis of survey data and review of research in South Asia (Paintal & Aguayo, 2016) found that most children (up to 98%) continue to be breastfed when they become sick. However, up to 49% of sick children are breastfed less frequently than usual and up to 75% of caregivers restrict the frequency, quantity, or quality of foods fed to sick children (Paintal & Aguayo, 2016). Common reasons given by caregivers for these practices include concerns that a sick child cannot digest breastmilk or some food types, that the breastmilk has become harmful to the child, and that the child has no appetite or refuses to feed. The study also finds evidence that feeding advice and counselling by health workers to caregivers of sick children are inadequate and in some cases detrimental.

Taken together, these papers indicate that efforts to improve the diets of young children should combine interventions to build the knowledge and skills of caregivers on recommended feeding practices with approaches to improve the access of households to affordable nutritious foods. A review of recent research in South Asia has shown that information, education, and counselling delivered by a range of well‐trained primary health care workers and community resource persons can improve the timeliness, frequency, diversity, and/or adequacy of complementary feeding (Aguayo, 2017). However, in some contexts, the impact of these interventions may be limited by the availability or affordability of nutritious foods (Aguayo, 2017), reaffirming the findings of the predictor analysis and the need to simultaneously address economic barriers.

## 6 AIMING HIGHER FOR MATERNAL AND CHILD NUTRITION IN SOUTH ASIA

The set of papers in this special issue bring together research and programmatic evidence on the trends, drivers, and inequalities on stunting, wasting, anaemia, and poor child feeding in South Asia, and the pathways to improving the growth, development, and well‐being of children and women in the region. Among others, the findings have the following five implications for the direction of future advocacy, policy, and programme actions.

### 6.1 First, the co‐occurrence of child stunting with wasting and anaemia in South Asia requires governments to address all forms of malnutrition in an integrated manner across the life cycle

Stunting, which has gained prominence under the Scaling Up Nutrition movement, is part of a wider nutrition crisis in South Asia's children that includes wasting, micronutrient deficiencies, and anaemia. In the past, there has been a tendency to address these different forms of malnutrition in isolation (Khara & Dolan, 2014) and with varying levels of intensity. However, they often affect the same children and share common risk factors, including the poor nutritional status of women and mothers, children's poor diets and feeding practices in the first 2 years of life, and socio‐economic deprivation, including household food insecurity and poverty. Policies and programmes should move away from siloed approaches and be realigned to addressing maternal and child malnutrition in all its forms.

### 6.2 Second, improving women's and children's diets is central to breaking the intergenerational cycle of malnutrition in South Asia

Although further improvements are needed, the region has made relatively good progress in improving breastfeeding practices. However, complementary foods and feeding practices remain unacceptably poor. The lower progress on complementary feeding compared with breastfeeding reflects the relative intensity of the policy and programme focus: a recent policy analysis in Bangladesh, India, Nepal, Pakistan, and Sri Lanka found that the policy landscape for complementary feeding is weaker than for breastfeeding, and lacks clarity in terms of interventions, approaches, and coordination among different sectors needed to improve the quality of complementary foods and feeding practices (Thow et al., 2017). Children's diets need much greater attention by all stakeholders concerned with the growth and development of South Asia's children and the competitiveness of national economies. It requires strategies to improve access to nutritious and affordable foods coupled with communication interventions for behaviour and social change, as physical, financial, and social constraints to optimal complementary feeding often coexist. This series of papers also reaffirms the close connect between the nutritional status of a mother and her children, and the need to tackle the dietary drivers of poor women's nutrition before and during pregnancy. In addition, there is need to press for policy and societal actions to raise the age at marriage and first pregnancy.

### 6.3 Third, a coordinated multisystem approach is needed to ensure families have all the inputs they need for children's healthy growth

The drivers of poor growth in early life—poor maternal nutrition, poor child diets, and poor water sanitation and hygiene (WASH)—cannot be fixed by one system alone. The food, health, and social protection systems should combine actions to improve the dietary intake of women and young children by increasing the availability of nutritious foods, alleviating financial barriers to the acquisition of these foods, and increasing the knowledge and skills of women and other caregivers to prepare and feed these foods hygienically. In addition, the WASH system needs to ensure the access to and use of safe water and sanitation services, and the education system should increase girl's access to primary and secondary education in contexts where they are deprived of educational opportunities. In practice, this requires coordination between sectors as well as between different levels of government.

### 6.4 Fourth, deliberate actions are needed to address the disparities and inequalities in child growth in early life

The disproportionate burden experienced by the most disadvantaged children and worsening inequalities between socio‐economic groups are pressing concerns for equitable and sustainable growth. Targeted efforts are needed to reach children, mothers, and communities at greatest risk of malnutrition. At the individual level, interventions should focus on children born small, children under 2 years of age, younger less experienced mothers, and poorer households. At the community level, communities with higher levels of anthropometric failure and lower access to maternal and child health and nutrition services should be prioritized. Evidence shows that focusing on community‐level interventions by trained health care workers and community volunteers can help address inequitable access to services.

### 6.5 Fifth, continued attention is needed in all countries to gather, analyse, and use data to assess progress and inform decisions

This includes the use of routine information systems and periodic surveys to gather data on anthropometric indicators, feeding practices, and the coverage of essential nutrition, health, and social protection services for children and women. In addition, studies and implementation research are needed to better understand the context‐specific barriers, enablers, and pathways to improving the access to maternal and child nutrition, health, and social protection services and the adoption of recommended nutrition behaviours and practices by caregivers and care providers.

## 7 CONCLUSIONS

The South Asia region bears a disproportionate burden of stunted children who experience worse health, cognition, and learning outcomes. These children are concentrated in the more socio‐economically disadvantaged households and often experience multiple forms of nutrition deprivation, including LBW, stunting, wasting, and anaemia. Accelerating progress in this context will require much greater attention to improving the nutritional status of women before and during pregnancy and to the diets of infants and young children in the first 2 years of life, while addressing their underlying drivers. A coordinated multisystem approach and actions to tackle the disparities and inequalities in child growth are needed. The series of papers in this special issue of Maternal & Child Nutrition present the evidence on these issues. It is our hope that they will support the efforts by all stakeholders concerned with the growth and development of South Asia's children and the sustainable development of its nations.

## CONFLICTS OF INTEREST

H. T. and V. M. A. are members of the United Nations Children's Fund (UNICEF). The authors alone are responsible for the views expressed in this publication and declare that they have no conflicts of interest.

## CONTRIBUTIONS

HT and VMA prepared the manuscript.

## ETHICAL STANDARDS DISCLOSURE

Not applicable.
